# Erratum: Stable and solubilized active Au atom clusters for selective epoxidation of *cis*-cyclooctene with molecular oxygen

**DOI:** 10.1038/ncomms15669

**Published:** 2017-08-11

**Authors:** Linping Qian, Zhen Wang, Evgeny V. Beletskiy, Jingyue Liu, Haroldo J. dos Santos, Tiehu Li, Maria do C. Rangel, Mayfair C. Kung, Harold H. Kung

Nature Communications 8 Article number: 14881 ; DOI: 10.1038/ncomms14881 (2017); Published: 03 28 2017; Updated: 08 11 2017

The original version of this Article contained an error in which the second affiliation ‘Chemical and Biological Engineering Department, Northwestern University, Evanston, Illinois 60208, USA’; was inadvertently switched with the third affiliation ‘School of Materials Science and Engineering, Northwestern Polytechnical University, Xi’an, Shaanxi 710072, China’.

Additionally, ‘Shaanxi’ was misspelt as ‘Shaansi’. This has now been corrected in both the PDF and HTML versions of the Article.

