# Supplementary material for: Stable and solubilized active Au atom clusters for selective epoxidation of cis-cyclooctene with molecular oxygen
Source: Nat Commun. 2017 Mar 28;8:14881. doi: 10.1038/ncomms14881 (PMC5379066; doi:10.1038/ncomms14881)
Supplement: Supplementary Information — Supplementary Figures, Supplementary Tables, Supplementary Methods and Supplementary References. [file ncomms14881-s1.pdf]

Supplementary Information

Supplementary Methods

1.1. Sources and Purification of Chemicals and Reagents:

AuCl (99.9%, Alfa Aesar), $AuCl_3$ (99.99%, Aldrich), decane (≥99%, Sigma Aldrich), *cis*-cyclooctene (95%, Alfa Aesar) *cis*-1,2-cyclooctanediol (99%, Aldrich), cyclooctanol (99%, Aldrich), cyclooctene oxide (99%, Aldrich), 1,5-cyclooctadiene(Aldrich, >99%), 1,3-cyclooctadiene (Aldrich, >95%) tetrahydrofuran (Aldrich> 99%), triphenylphosphine(>98.5%, Sigma Aldrich), EM Quant peroxide test strips, KI (Sigma Aldrich), fumed silica (CAB-O-SIL-90, Cabot Corporation), polymethylhydrosiloxane (Sigma Aldrich) were reagents, solvents and standards used in this work.

The manufacturer-added stabilizer (100 to 200 ppm irganox 1076) in cyclooctene was removed using 3 M KOH treatment at room temperature followed by separation and repeated washing with milli-Q water.[1] Sometimes this procedure was not adequate to remove all impurities, then distillation of COE was conducted using an oil bath at around 180 ^oC while collecting the sample at 145 ^oC. The first fraction of the distillate was discarded.

1.2. Catalyst Synthesis

Au/SiO_2-A was prepared using $Au(en)_2Cl_3$ precursor (en = ethylenediamine), similar to the procedure of Zhu et. al[2]. 10mL of ethanol was added to 0.5g of $HAuCl_4.3H_2O$ dissolved in 2 mL of water and the solution was stirred for 5 min. Then 0.23 mL of en was added to result in an en/Au ratio of 2.65, and the mixture was stirred for 10 min and the solid was filtered and washed with 300 mL of ethanol. Then a quantity of 4.2 mM of this solution at 40 ^oC was added to fume silica (CABO-SIL-L90) such that the Au loading was 2.2 wt.%. The pH of the solution was adjusted to 9 by dropwise addition of 0.75M en solution and the solution was stirred for 2h at room temperature before filtration and washing with 500 mL of water. It was re-dispersed in 200 mL of water at 40 ^oC, and then filtered and washed with 500 mL of water. After calcination, the resulting powder was light yellow in color, and had a Au loading of 1.2 wt.%.

Au/SiO_2-B was synthesized similar to our previously published method.[3] Briefly, poly(methylhydro)siloxane with trimethylsiloxy termination (PMHS) was partially oxidized (~50%) with water over Pd/ carbon catalysts. The resulting silanol groups were further reacted with N-methyl-aza-2,2,4-trimethylsilacyclopentane to form amine functionalized PMHS. Then Au(THT)Cl was added to the amine functionalized PMHS (amine/Au = 10) and stirred for 0.5h before the introduction of (CABO-SIL-L90). The final Au loading of the sample was 0.9 wt.%. There were two different samples of Au/SiO_2-B1 and B2. For one preparation, the Au nanoparticle size was around 0.9 nm and for the other it was around 4.5 nm and they differed in the degree of washing and dryness before calcination. Since Au/SiO_2-B was prepared with siloxane polymer, Au particles would be partially encapsulated by thin islands of SiO_2 after calcination.

Calcination procedure was the same for both Au/SiO_2-A and Au/SiO_2-B. The catalyst was placed in a U-tube and O_2/O_3 mixture was flow through it at ~300 mL/min. The temperature was increased at 0.12^oC min until 150 ^oC and held at that temperature for 0.5h. After calcination, the average Au particle size was around 2 nm for Au/SiO_2-A.

AuCl and $AuCl_3$ were stored in the dark in a jar containing drierite and used as is.

1.3. Characterization

1.3.1. Emission and excitation spectra. The spectra of the reaction solutions were analyzed using Photon Technology International Model QM-2. The emission spectrum was collected by first determining the wavelength of the excitation light that would yield the maximum emission intensity. Once determined, then the emission spectrum was collected using this excitation wavelength. Excitation spectrum was generated by monitoring the fluorescence emission at the wavelength of the emission peak while varying the excitation wavelength. When sample was diluted, HPLC grade THF or ethanol was used.

1.3.2. ICP: Au solution concentration after a reaction was determined using Thermo iCAP Q Inductively Coupled Plasma Mass Spectrometer (ICP-MS). Before the measurement, the organic in 1 mL of the sample was burnt at 850 ^{o}C for 4 h in a muffle furnace. Then, 0.2 mL concentrated HNO_3 and 0.2 mL concentrated HCl were added to dissolve the Au species. After that, the solution was transferred to a volumetric flask and diluted to a total volume of 10 mL with Milli-Q water.

1.3.3. TEM:
High-angle annular dark-field scanning transmission electron microscopy (HAADF-STEM) images were obtained on a JEOL ARM-200F aberration-corrected STEM (AC-STEM) operated at 200 kV with a nominal spatial resolution of 0.08 nm in the STEM mode. The TEM/STEM samples were prepared by dipping the lacey carbon covered copper TEM grid directly into the ethanol diluted filtrate solution that contained the Au nanoclusters. After drying, the filtrate formed a thin polymer film that hung over the holes of the lacey carbon film. The filtrate coated TEM grid was inserted into the microscope for observation.

1.4. Catalytic test

1.4.1. General procedure: The catalytic oxidation of *cis*-cyclooctene (COE) over gold based catalysts was conducted in a set-up shown in Supplementary Figure 16 in the absence of light. 10 mL of COE, 1 mL n-decane (internal standard), Au catalyst and a teflon-coated magnetic stirrer were loaded into a 50 mL three-neck glass cylindrical shaped flask. Then the mixture was sonicated for 10 mins in the dark and stirred briefly and then placed in an oil bath preheated at 100 ^{o}C. The reaction mixture was stirred until the temperature reached 100 ^{o}C and then ultra high purity O_2 (Airgas company) was introduced using a fine frit glass disperser tube (Chemglass Sciences) and time zero corresponds to the time of O_2 introduction. A reflux condenser maintained at -5° C with a Fisher Scientific ISOTEMP 6200R35 refrigerated/heated bath circulators was used to decrease the evaporation rate of the reagents. Aliquots of the reaction mixture were taken at different time intervals, diluted with anhydrous THF (≥99%, Sigma Aldrich) and frozen until the time of analysis. For GC analysis, the samples were brought to room temperature and analyzed using an Agilent 6890 GC equipped with a FID connected to Agilent J and W DB-624 capillary column (30 m × 0.25 mm × 0.25 μm). The identification of the products was primarily by GC-MS spectrometry (Agilent GC-7890A, MS-5975). Product

identities were further confirmed with ^1H and ^{13}C NMR. It should be noted that under our GC analysis conditions cyclooctene hydroperoxide was not detected. However, it was detected using ^1H NMR. Sensitivity factors of the different products were calibrated with commercially available standards when possible. Cyclooctanol and cyclooctanone were used as substitutes for cyclooctenol and cyclooctenone.

1.4.2. Three types of catalysts, Au/SiO_2, $AuCl_3$ and AuCl were tested.

Au/SiO_2: 80 mg of 1 wt. % Au/SiO_2 was placed in a three necked flask fitted with a fine frit glass disperser tube and a condenser. 10 mL of stabilizer-free COE and 1 ml of the internal standard decane were then introduced and stirred vigorously with a magnetic stir bar. Then the three necked flask was lowered into a preheated oil bath at 100 ^oC. At the termination of the reaction, the catalyst was hot-filtered using a 0.20 μm syringe filter.

$AuCl_3$ and AuCl: 6 mg of AuCl or 8 mg of $AuCl_3$ was placed in the reactor with COE and decane internal standard. Stabilizer of cyclooctene was not removed. In experiments when cyclooctane diol was included in the reaction mixture, the diol was first mixed with the solution of COE and decane. Then, the mixture was heated at 100 ^oC in the oil bath for 3 min to dissolve the diol completely. After that, AuCl was added and the solution was stirred for 20 min at room temperature and then the standard procedure for COE was resumed.

1.4.3. Conversion and selectivity calculation: GC area ratio of molecule of interest to the internal standard decane was used in the conversion and selectivity calculations. The boiling points of the different chemicals vary as is shown in Supplementary Table 8 with COE having significantly lower boiling points than the others. Since some of the reactions were conducted over many hours it may be essential to correct for the evaporation rate of COE. COA was chosen as a surrogate molecule for COE in the evaporation calibration as it is non-reactive in air and its boiling point differs from COE by only 3-4 degrees. Supplementary Figure 2 shows the (COA) /decane ratio as a function of time on stream at 100 ^oC. Corrections for evaporation based on the slope of Supplementary Figure 2 may be over-estimated at high conversions of COE as the vapor pressure of COE decreased when its mole fraction in solution decreased. Therefore $COE_{avg,t}$ is the average of the values of COE, corrected and not corrected for evaporation at time t. (Eq. 1) This value was used for conversion and rate calculations. The low evaporation rate under our reaction conditions indicates that corrections for the evaporation of higher boiling products are not necessary.

$COE_{avg,t} = [COE_t + COE_{evap,t} + COE_t]/2$ (1)

$COE_{evap,t} = (COE_{inital} * t * 0.0072)$ where 0.0072 is the slope value from Fig. 2 (2)

and $COE_{initial}$ is COE/decane at time = 0

Conversion (%) at time t = $100*[COE_{initial} - COE_{avg,t}]/COE_{inital}$ (3)

Selectivity of product n (%) = $I_n * 100 / \sum_i I_i$ (4)

where I_i is the GC area for product i corrected for its sensitivity factor relative to COE.

Yield (%) of product n = (selectivity for product n/100)*(conversion/100)*100 (5)

1.4.4. Glassware cleaning procedure.

Gold and organics were deposited on the disperser and sometimes on the reactor walls. After the reaction was completed, ethanol was added to the reactor and disperser assembly and sonicated to remove the organics. This procedure was repeated three times. To remove the deposited gold, the disperser was immersed in aqua regia in the reactor overnight. After rinsing with water and then ethanol, it was washed with distilled water three times and the assembly was dried in an oven. Before the experiments, all the glasswares were dried again with a heat gun. Control experiment was conducted with reaction-used glasswares that were cleaned with the above procedures. The control experiment was conducted with COE with stabilizer removed and no Au present at 100 ^{o}C and with O_2 bubbling through the disperser at 30mL/minute, conditions similar to the catalytic run but with no Au catalysts. There were no reactions up till 11h, but 24% conversions were observed after 23h. Since most of our experiments had duration shorter than 23h, the low level of auto-oxidation of COE does not affect the data.

1.5. Titration for peroxides.

Triphenylphosphine (5 uM in dichloromethane or ethanol) was added to 0.1 mL of the sample continuously until the peroxide test paper (EMD chemicals Inc.0.5-25 ppm range) color turned colorless indicating the absence of peroxide. Since peroxide test paper may not be sensitive to polymeric hydroperoxide, KI and starch test was also used to verify the absence of polymeric hydroperoxide. 1 mL of a solution of 0.09 M KI and 0.011 wt% starch indicator was added to 1 ml of a sample that been treated with triphenylphosphine. After sonicating for 10 mins, no dark blue color was observed, indicating peroxides had been completely removed by triphenylphosphine.

1.6. Rates at 50% conversion for filtrates obtained through consecutive dilutions.

For reaction with Au/SiO_2-A, 6 nmol of Au was in the original solution. We assumed after 29-fold dilution, the contribution of Au to be insignificant, then the contribution of Au towards the auto-catalytic phase at 50% conversion was (5.3-3.5)/6=0.33 mmol $(\text{nmol Au-h})^{-1}$. For Au/SiO_2-B catalyst, the initial Au in the filtrate was much lower and therefore, its contribution to the reaction at 50% was lower. Assuming that by the first dilution, Au was not contributing much, then the contribution = (3.9-3.3)/1.57 = 0.38 mmol $(\text{nmol Au}\cdot\text{h})^{-1}$.

1.7. Cyclohexene oxidation with O_2 using filtrate derived from COE oxidation in the presence of Au/SiO_2-A.

Standard COE epoxidation was conducted in the presence of Au/SiO_2-A. Triphenylphosphine was added to the after reaction solution obtained by hot filtration in order to remove the hydroperoxide. Then 2 mL of this solution was added to 8 mL of cyclohexene and 0.2 mL of

decane. The solution was placed in an oil bath heated to 60 ^{o}C and then 30mL of O_2 was bubbled into the reaction solution.

1.8. Experiments using *t*-BuOOH as initiator.

300 µl of 5.5 M *t*-BuOOH in decane (1:46.5 *t*-BuOOH: COE) was added to 10 mL COE and 1 mL internal standard decane but with no Au catalyst and the reaction was conducted under standard reaction conditions.

Supplementary Tables

Supplementary Table 1. Product selectivities over Au/SiO_2, AuCl and $AuCl_3$ catalysts.

Catalyst	Conversion (%)	Selectivity (%)[a]			
		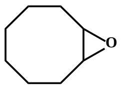	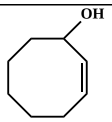	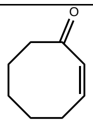	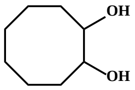
Au/SiO_2–A	47	79	6	6	3
Au/SiO_2-B2	46	81	5	5	2
$AuCl_3$	46	79	7	5	3
AuCl	45	82	6	4	2
AuCl + diol (1:50)	47	80	5	7	2

[a]: There are several small unidentified peaks whose total GC areas are about 5-7% of the products at 50% conversions. There appeared to be more such peaks when diol was added deliberately to the reaction solution. At low conversions (10-12%) carbon balance was within ± 0.6-1.5%. At 50% conversion, carbon balance was within ± 10%.

Supplementary Table 2. Reaction rates at 50% conversion for experiment using different Au precursors.

Au source	Rate at 50% conversion, mmol/h
Au/SiO_2-A	5.3±0.2
Au/SiO_2-B2	3.9±0.2
AuCl	3.5±0.2

Supplementary Table 3. Dependence of induction period and soluble Au in solution on the diol/Au in the COE reaction solution using AuCl as the Au source.

Diol/Au	100	50	25	0
Induction period (h)	2.9	3.8	6.0	9.4
Au ng ml^{-1}	1930	374	95	11

Supplementary Table 4. Effects of solubilized Au concentration and triphenylphosphine addition on the initial cyclooctene consumption rate.

Exp.	Sample[a]	Initial rate,[b] mmol h^{-1}
1	5.4 times diluted stock Au filtrate + 1.0 eq. PPh_3	2.1±0.3
2[c]	157 times diluted stock Au filtrate + 1.0 eq. PPh_3	0.10±0.05
3	5.4 times diluted stock *t*-BuOOH filtrate + 1.0 eq. PPh_3	~0.00

[a] Stock Au filtrate was derived from a reaction using Au/SiO_2-A, and contained ~100 ng mL^{-1} solubilized Au. Stock *t*-BuOOH was derived from a reaction using *tert*-butyl peroxide as initiator. An amount of PPh_3, 1.0 equivalent of hydroperoxide present in the reaction, was added to each of these solutions prior to the experiment. [b] Initial cyclooctene consumption rate. [C] The procedure for experiment 2 is shown in the diagram below. 2 mL of the reaction mixture at the end of Exp. 1 was used to initiate a second reaction. Then 2 mL of the product mixture of this second reaction was mixed with 1.0 eq. PPh_3 to initiate Exp. 2.

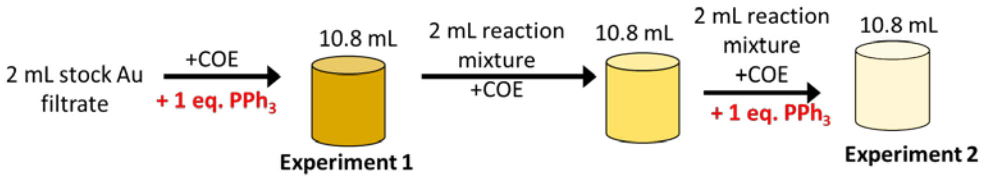

Supplementary Table 5. Reaction rates at 50% conversion for experiments with consecutive dilution of filtrates derived from Au/SiO_2.

Experiment	Rate at 50% conversion, mmol/h	Au ng/ml
First reaction with Au/SiO_2-A	5.3±0.2	119[a]
First filtrate experiment (5.4 fold dilution)	4.6±0.2	22[b]
Second filtrate experiment (29 fold dilution)	3.5±0.2	4[b]
First reaction with Au/SiO_2-B	3.9±0.2	31[a]
First filtrate experiment (3.6 fold dilution)	3.3±0.2	8.7[b]
Second filtrate experiment (13 fold dilution)	3.3±0.2	2.4[b]

a: by ICP. b: calculated from dilution.

Supplementary Table 6. Excitation and Emission spectra calculated using the Jellium model[4].

cluster size	Energy eV	λ nm
1	5.50	225
2	4.37	284
3	3.81	325
4	3.46	358
5	3.22	385
6	3.03	410
7	2.88	431
8	2.75	451
9	2.64	469
10	2.55	486

Supplementary Table 7. Shift of the sharp instrument artifact peak with respect to the incident light wavelength, using water as the sample.

$\lambda_{excitation}$ (nm)	Peak of artifact (nm)	$\Delta\lambda_{excitation}$ and peak (nm)
280	297	17
350	385	35
355	392	37
375	418	43
400	452	52
425	486	61

Supplementary Table 8. Boiling point of reactants and products (all of the data with the exception of 2-cycloocten-1-ol were at atmospheric pressure and obtained directly from the web).

Chemical	Boiling Point ^{o}C
cis-Cyclooctene (COE)	145-146
Cyclooctane (COA)	149
Decane	174
Cyclooctane epoxide	189-190
2-Cycloocten-1-nol	105-106 (23 Torr)[5]
Cyclooctanone	196
Cyclooctanol	203
Cyclooctanediol	265

Supplementary Figures

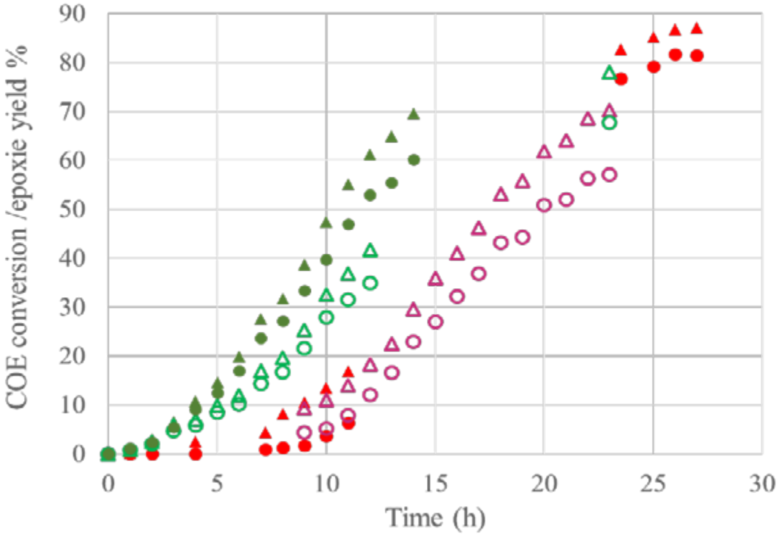

Supplementary Figure 1. COE conversions (circles) and cyclooctane epoxide yields (triangle) over different Au/SiO_2 catalysts. Open and filled green symbols are data obtained with duplicate preparations of Au/SiO_2-A. The TEM average Au particle size of the sample represented by the open symbols was 2.3 nm. Open and filled red and burgundy data points were collected using Au/SiO_2-B1 and Au/SiO_2-B2 of an average Au particle size of 0.9 nm and 4.5 nm, respectively.

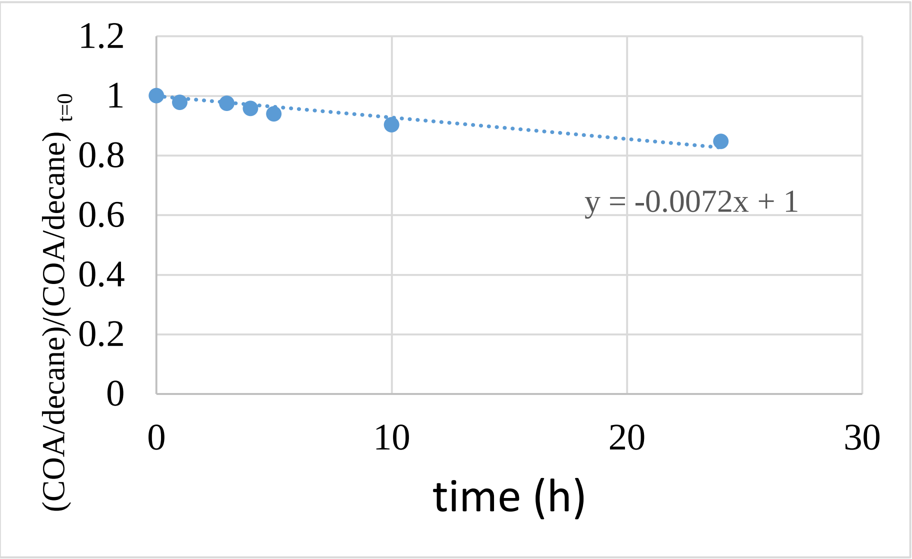

Supplementary Figure 2. Normalized cyclooctane/decane (COA/decane) ratio as a function of time at 100°C as an indicator of the evaporation rate of COE.

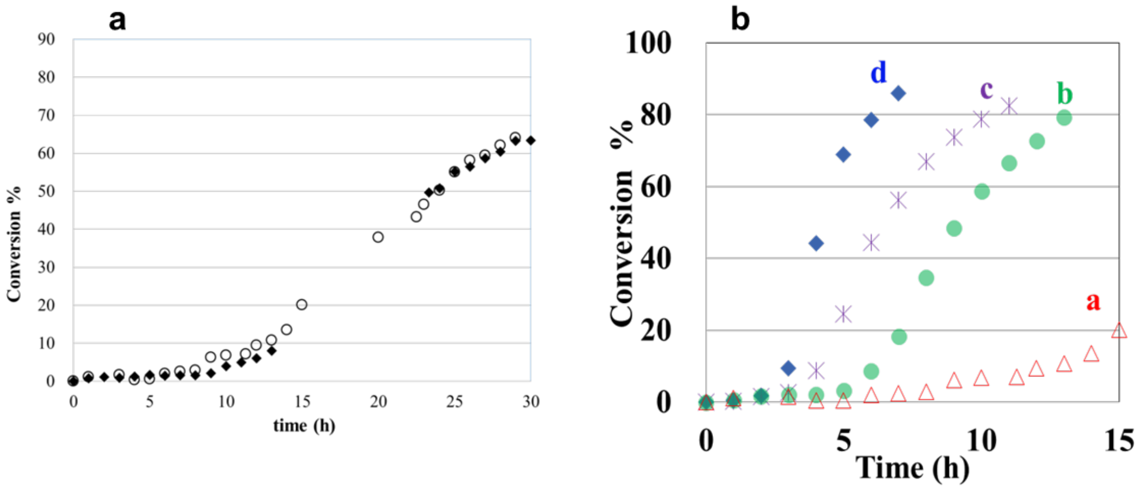

Supplementary Figure 3. Effect of adding cyclooctanol. a: Reaction profile of COE oxidation using AuCl without (o) and with (♦) added cyclooctanol (cyclooctanol/Au = 100). b: Comparison of time on stream profile of AuCl catalyzed COE reactions in the presence of cyclooctanediol. Cyclooctanediol:Au molar ratio: (d) 100:1, (c) 50:1 (b) 25:1 and (a) 0:1.

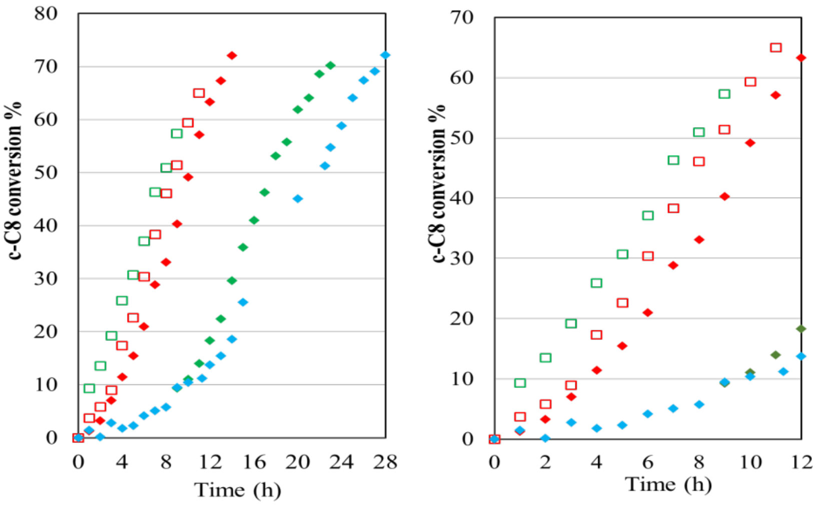

Supplementary Figure 4. Comparison of reaction profiles for reaction using various Au precatalysts versus those using the corresponding filtrates. Diamonds are runs using Au precatalysts and square using filtrates. Red: Au/SiO_2-A and 5.4 times diluted filtrate. Green: Au/SiO_2-B2 and 3.6 times diluted filtrate. Blue: AuCl. Right panel expanded the early reaction times.

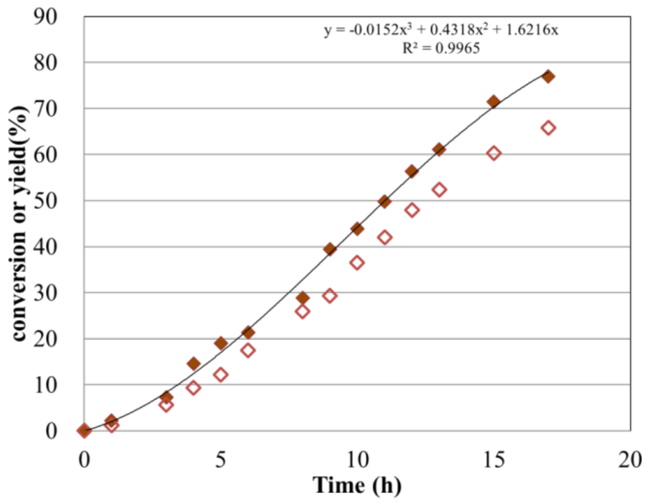

Supplementary Figure 5. COE conversion and epoxide yield for reaction started using *t*-BuOOH as initiator.

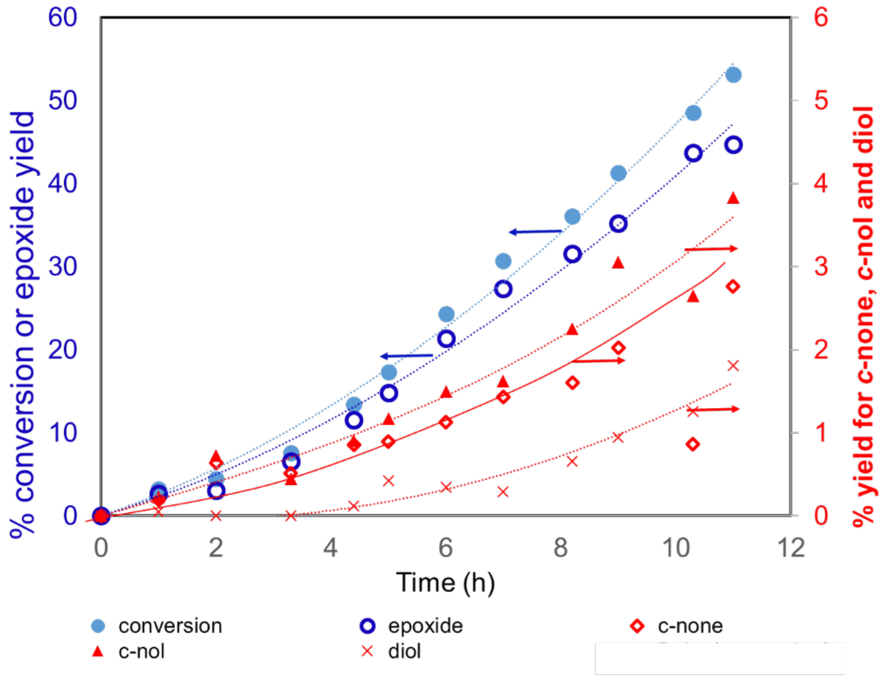

Supplementary Figure 6. Cyclooctene conversion and yields of epoxide, cyclooctenone (c-none), cyclooctenol (c-nol), and cyclooctanediol (c-diol) as a function of reaction time, for a reaction with filtrate derived from Au/SiO_2. An amount of triphenylphosphine equivalent to 1.05 equivalent of hydroperoxide was added to the filtrate.

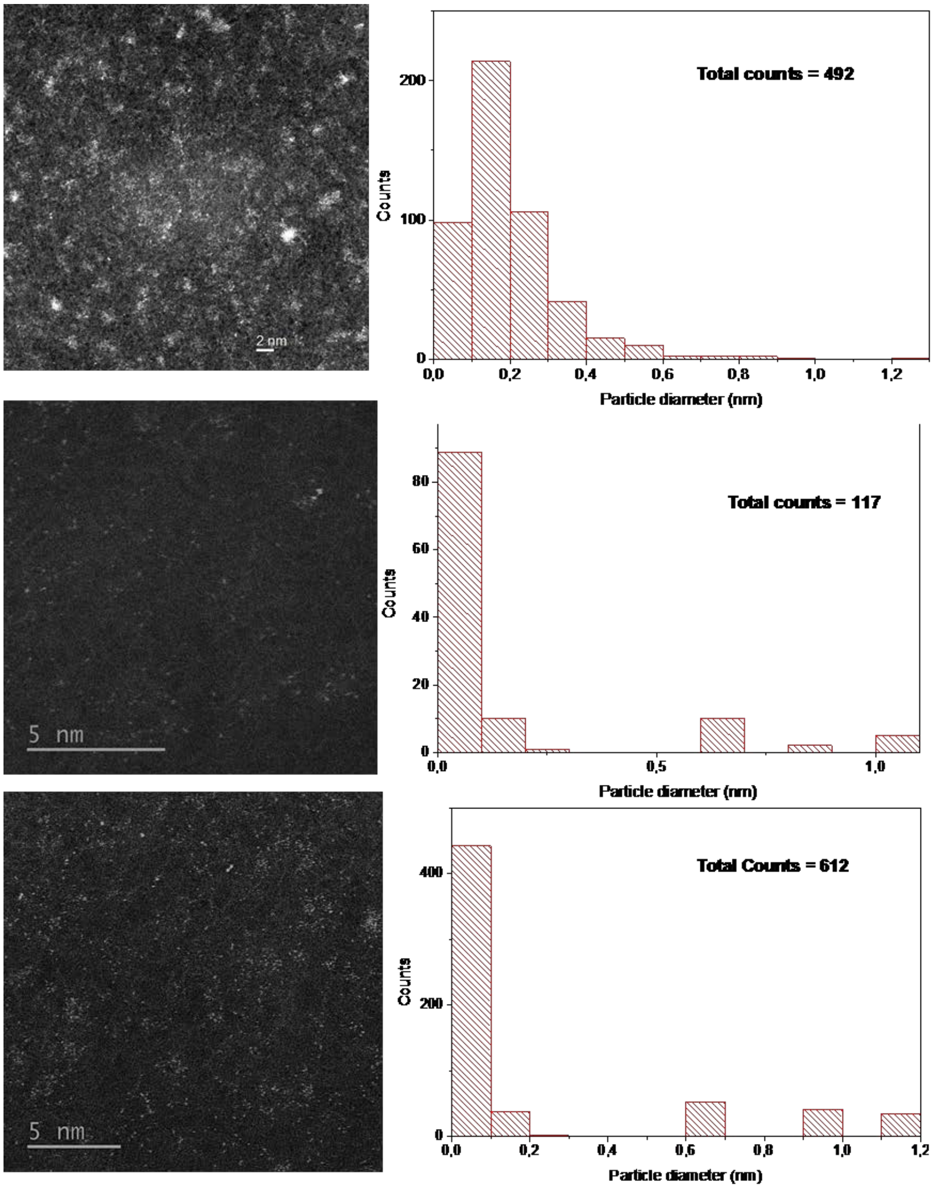
2 nm
Total counts = 492
Counts
Particle diameter (nm)
5 nm
Total counts = 117
Counts
Particle diameter (nm)
5 nm
Total Counts = 612
Counts
Particle diameter (nm)

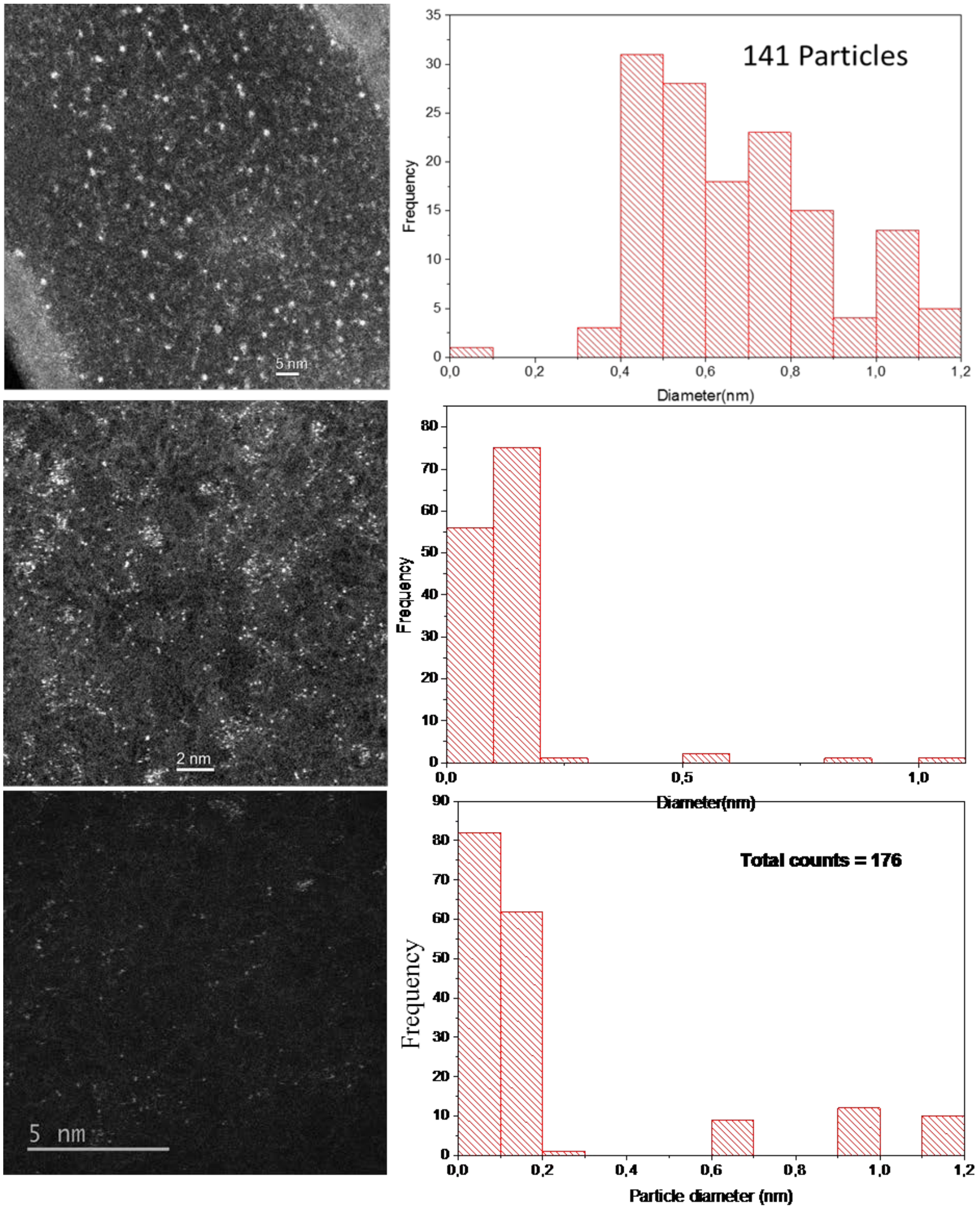

Supplementary Figure 7. Analysis of different regions of the TEM images of filtrate from Au/SiO_2-A.

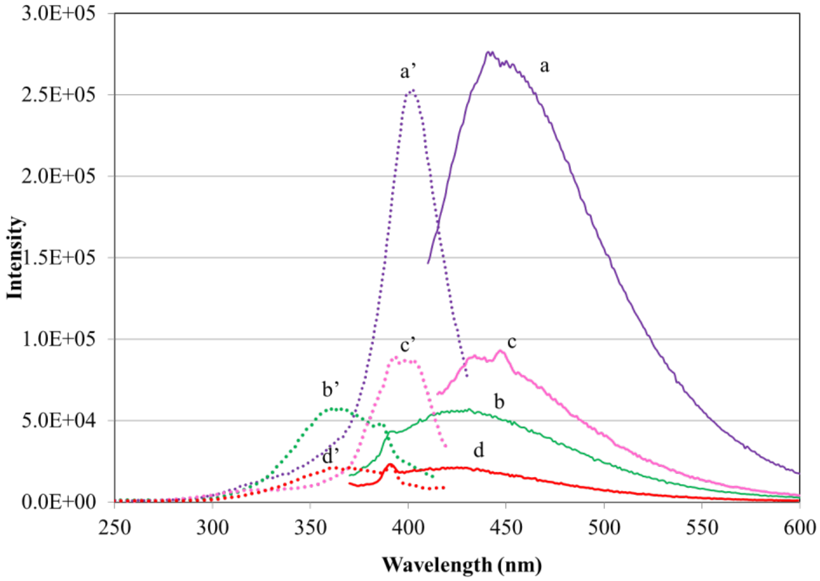

Supplementary Figure 8. Comparison of excitation and emission spectra of filtrate derived from Au/SiO_2-A and *t*-BuOOH reactions. Excitation spectra are dotted curves and emission spectra are solid curves. Au/SiO_2-A filtrate (a' and a) and Au/SiO_2-A filtrate diluted 10 times (b' and b); *t*-BuOOH filtrate (c' and c) and *t*-BuOOH filtrate diluted 10 times (d' and d). Excitation spectra monitored at $\lambda_{emission}$ =450 nm (a'), 425 nm (b') 430 nm (c' and d'). Emission spectra: $\lambda_{\text{Excitation}}$ = 405 nm (a and c); 360 nm (b and d).

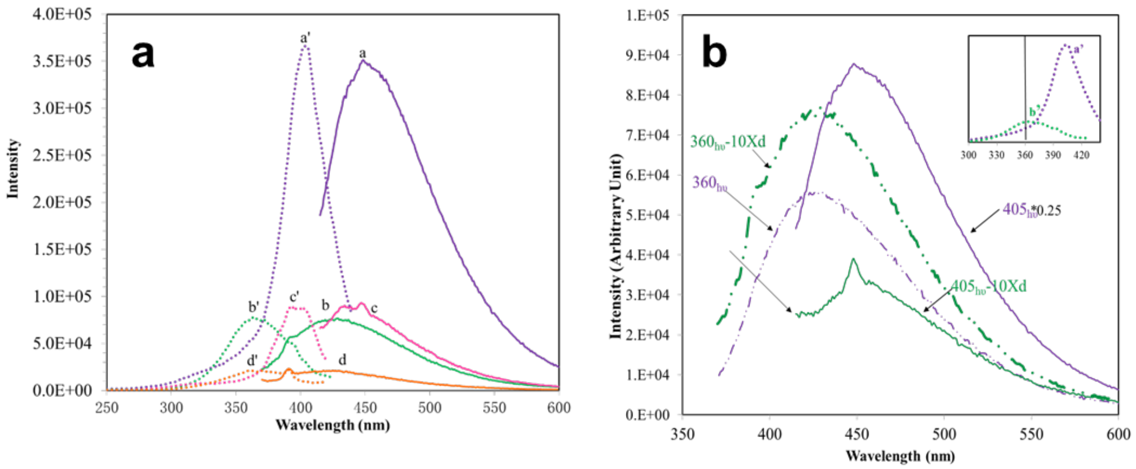

Supplementary Figure 9. Comparison of excitation and emission spectra of filtrate derived from AuCl and *t*-BuOOH reactions. Excitation spectra are dotted curves and emission spectra are solid curves. a: AuCl filtrate (a' and a) and AuCl filtrate diluted 10 times (b' and b); *t*-BuOOH filtrate (c' and c) and *t*-BuOOH filtrate diluted 10 times (d' and d). Excitation spectra; monitored at $\lambda_{emission}$ =450 nm (a' and c'), 435 nm (b' and d'). Emission spectra : $\lambda_{\text{Excitation}}$ = 405 nm (a and c) ; 360nm (b and d). b: Emission spectra of filtrate derived from AuCl (purple) and ten times diluted filtrate (green curves). Solid lines: $\lambda_{excitation}$ =405 nm; dash lines: $\lambda_{excitation}$ =360 nm. Inset shows the excitation spectra of the undiluted and 10 times diluted filtrate.

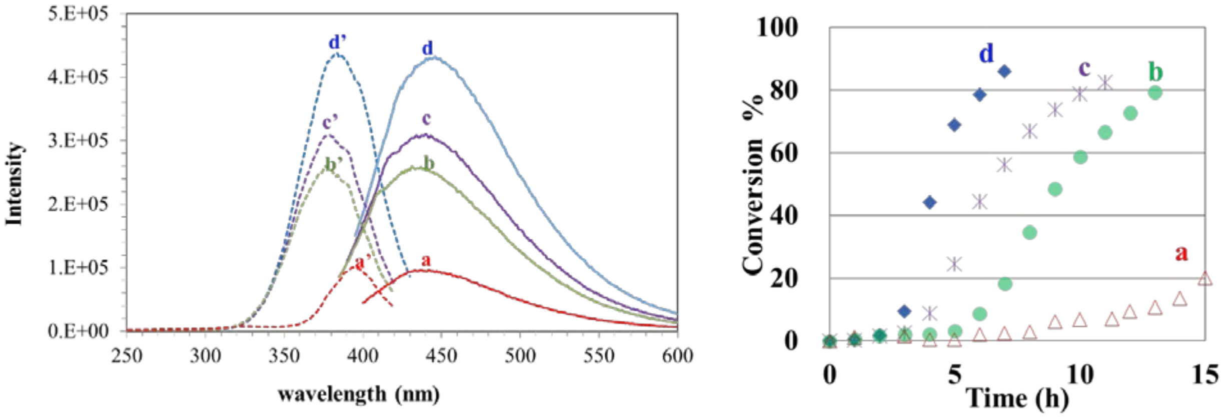

Supplementary Figure 10. Excitation and emission spectra of filtrate obtained from COE reactions with AuCl and diol. Diol/Au mole ratio =0 (a), 25(b), 50(c), 100(d). Excitation wavelengths are 390, 375, 380 and 385 nm for curves a,b,c and d. Curves a'- d' are collected by varying the excitation wavelength and monitoring the intensities at 430nm, for a'-c' and 440 nm for d'.

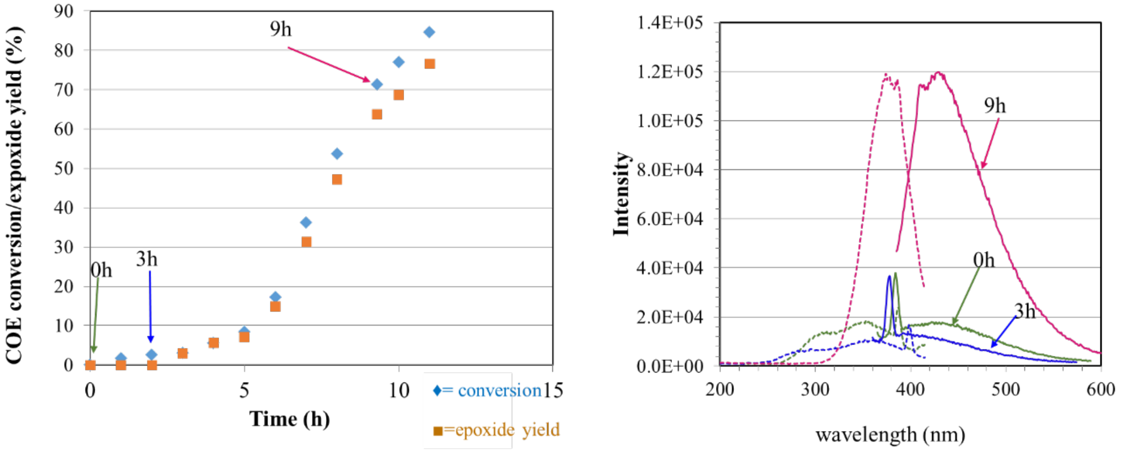

Supplementary Figure 11. Reaction profile and optical spectra of COE reaction solution as a function of reaction time. Left panel shows the COE conversion and epoxide yield in a reaction conducted using AuCl as the precatalyst and with added diol such that diol/Au=50. On the right panel are the excitation (dotted lines) and emission spectra (solid lines) collected at the indicated times of reaction. Solutions were diluted 10 fold with THF for spectroscopy. $\lambda_{excitation}$ = 255, 350 and 375 nm for 0, 3 and 9h emission spectra. $\lambda_{emission}$ = 425, 440 and 425 nm for 0, 3 and 9h, respectively. Very sharp peaks are instrumental artifacts.

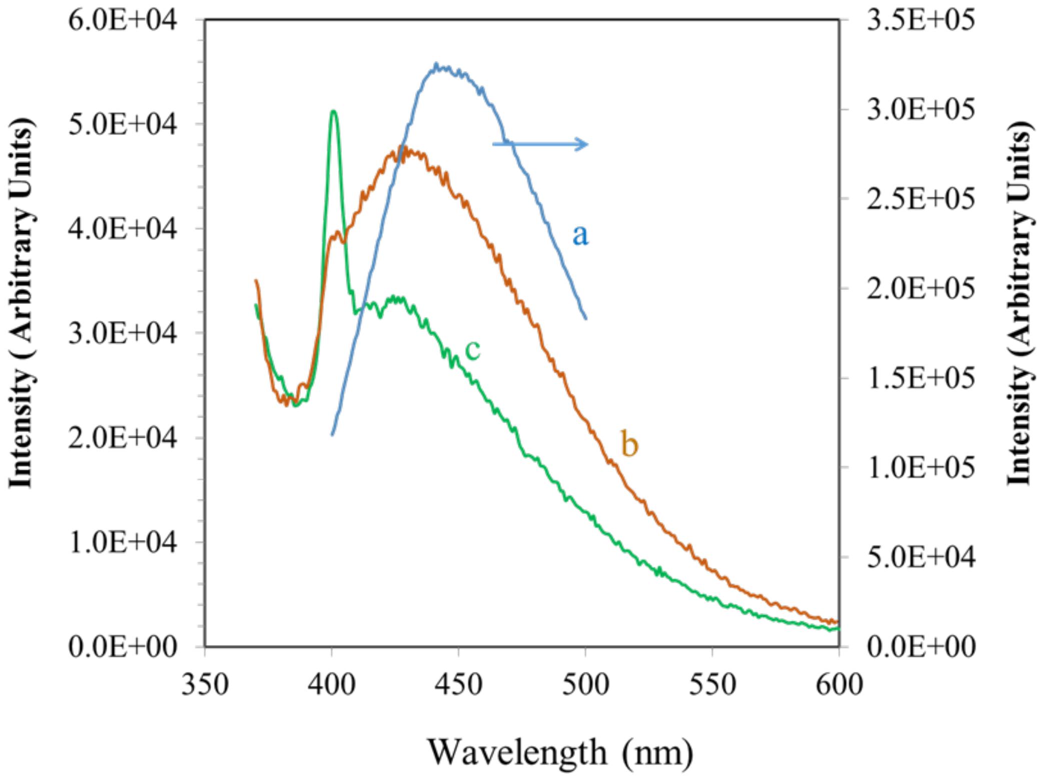

Supplementary Figure 12. Effect of solvents on the optical spectra of filtrates. Emission spectra of filtrate derived from Au/SiO_2-A (a) and the same filtrate diluted 12 times with ethanol (b) and COE (c). $\lambda_{excitation}$ = 390 nm (a) and 360 nm for b and c.

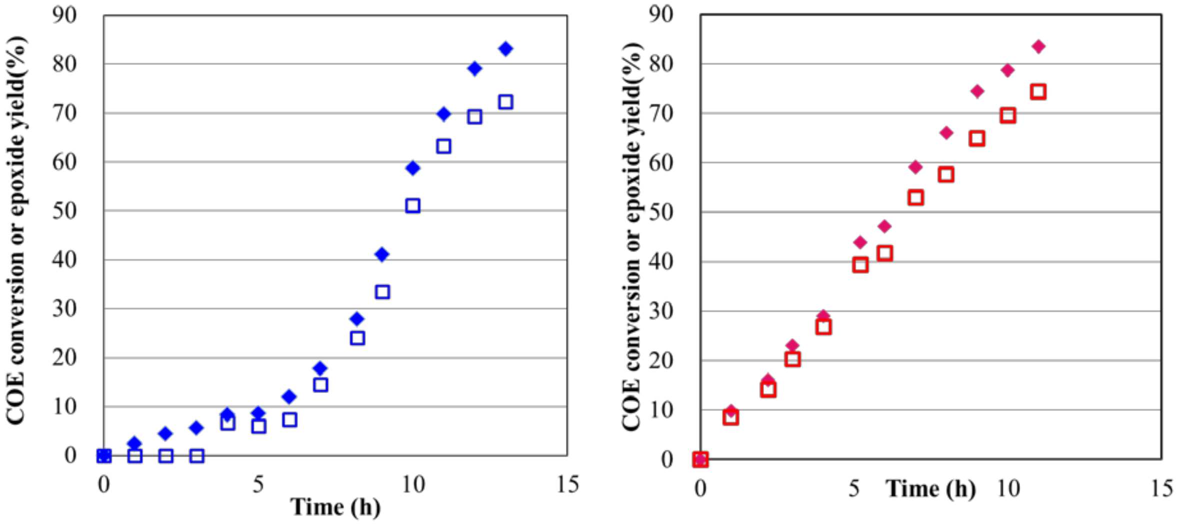

Supplementary Figure 13. COE oxidation with $PtCl_4$ in the presence of cyclooctanediol (diol/Pt=50) using O_2.COE conversions (diamond) and epoxide yields (square). $PtCl_4$ catalyzed COE oxidation in the presence of diol (diol/Pt=50) (left panel, blue). 2mL of the first filtrate was diluted with 8 ml of COE and 0.8 mL decane. A second oxidation reaction was conducted and the data are shown in red in the right panel.

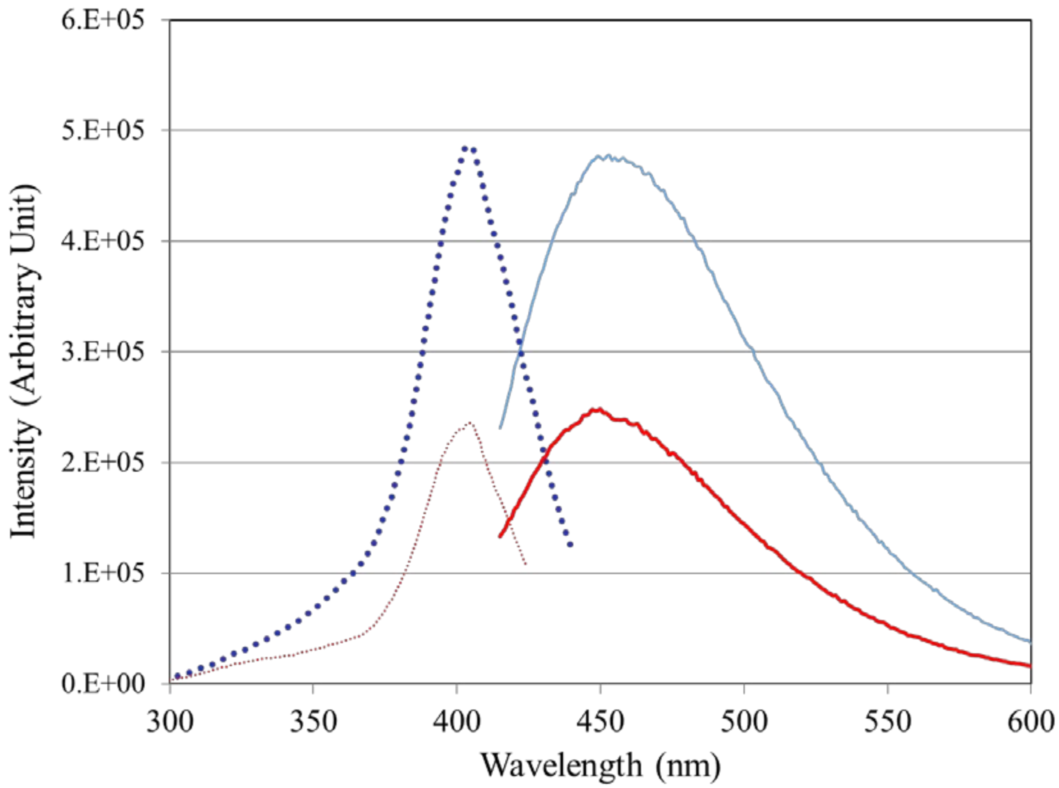

Supplementary Figure 14. Excitation (dotted line) and emission (solid line) spectra of filtrates from: (blue) $PtCl_4$ catalyzed COE oxidation in the presence of diol (diol/Pt=50); (red) reaction using 2 mL of the first filtrate added to fresh COE.

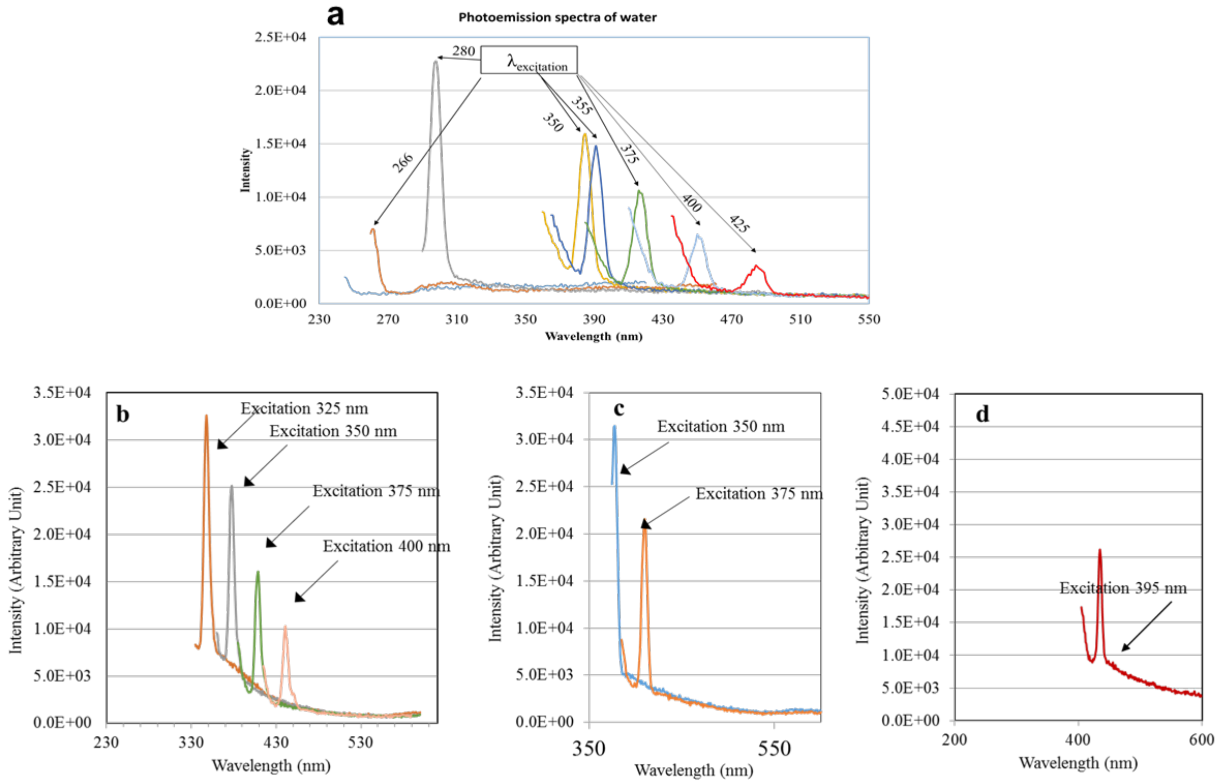

Supplementary Figure 15. Background spectra in the wavelength region of interest collected using: a: water; b: ethanol, c: HPLC grade THF, and d: cyclooctane epoxide. Sharp peaks are artifacts of instrument and not related to the solution. The spectra of the solvents used in spectral dilution and the product cyclooctane epoxide were featureless in the emission region of the Au clusters.

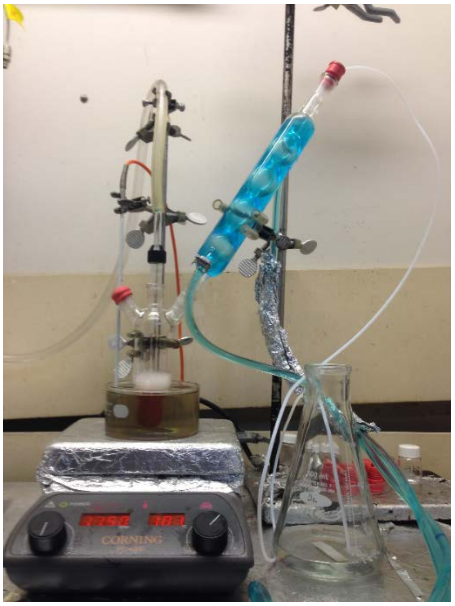

Supplementary Figure 16. Setup for the catalytic oxidation of c-C_8 with gold-based catalysts.

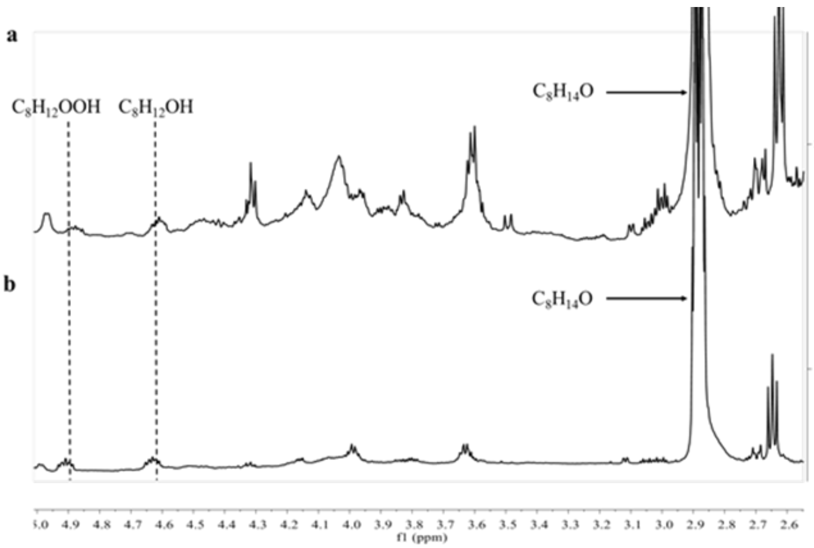

Supplementary Figure 17. ^{1}H NMR of reaction mixture. The spectra showed the presence of cyclooctene hydroperoxide in AuCl oxidation of COE. a is 29 h and b is 19h after the start of the reaction.

Supplementary References

1. Alshammari, H.; Miedziak, P. J.; Davies, T. E.; Willock, D. J.; Knight, D. W.; Hutchings, G. J., Initiator-free hydrocarbon oxidation using supported gold nanoparticles. *Catal. Sci. Technol.* **2014,** *4* (4), 908-911.
2. Zhu, H.; Ma, Z.; Clark, J. C.; Pan, Z.; Overbury, S. H.; Dai, S., Low-temperature CO oxidation on Au/fumed SiO2-based catalysts prepared from Au(en)2Cl3 precursor. *Appl. Catal., A* **2007,** *326* (1), 89-99.
3. Wang, Z.; Beletskiy, E. V.; Lee, S.; Hou, X.; Wu, Y.; Li, T.; Kung, M. C.; Kung, H. H., Amine-functionalized siloxane oligomer facilitated synthesis of subnanometer colloidal Au particles. *Journal of Materials Chemistry A: Materials for Energy and Sustainability* **2015,** *3* (4), 1743-1751.

4. Zheng, J.; Zhang, C.; Dickson, R. M., Highly Fluorescent, Water-Soluble, Size-Tunable Gold Quantum Dots. *Phys. Rev. Lett.* **2004,** *93* (7), 077402/1-077402/4.
5. Treboganov, A. D.; Astakhova, R. S.; Kraevskii, A. A.; Preobrazhenskii, N. A., Macrocyclic compounds. IV. New synthesis of cyclooctanone. *Zhurnal Organicheskoi Khimii* **1966,** *2* (12), 2178-81.
